# Cooperative benefit for the combination of rapamycin and imatinib in tuberous sclerosis complex neoplasia

**DOI:** 10.1186/2045-824X-4-11

**Published:** 2012-07-05

**Authors:** Baskaran Govindarajan, Laura Willoughby, Hamid Band, Adam S Curatolo, Emir Veledar, Suephy Chen, Michael Y Bonner, Martin-Garrido Abel, Marsha A Moses, Jack L Arbiser

**Affiliations:** 1 Department of Dermatology, Emory University School of Medicine; Winship Cancer Institute and Atlanta Veterans Administration Hospital, WMB 5309, 1639 Pierce Drive, Atlanta, GA, 30322, USA; 2 Eppley Institute for Research in Cancer and Allied Diseases, UNMC-Eppley Cancer Center, University of Nebraska Medical Center, Omaha, USA; 3 Vascular Biology Program, Children's Hospital Boston, Department of Surgery, Children's Hospital Boston and Harvard Medical School Karp Family Research Laboratories, Boston, MA, USA; 4 Department of Cardiology, Emory University School of Medicine, Atlanta, USA

## Abstract

Tuberous sclerosis (TS) is a common autosomal-dominant disorder characterized by tumors of the skin, lung, brain, and kidneys. Monotherapy with rapamycin however resulted in partial regression of tumors, implying the involvement of additional pathways. We have previously implicated platelet-derived growth factor-BB in TS-related tumorigenesis, thus providing a rationale for a combination of mTOR/PDGF blockade using rapamycin and imatinib. Here, we test this combination using a well-established preclinical model of cutaneous tumorigenesis in TS, tsc2ang1 cells derived from a skin tumor from a mouse heterozygous for tsc2. Treatment of tsc2ang1 cells with a combination of rapamycin and imatinib led to an inhibition of proliferation compared with either vehicle treatment or treatment with rapamycin or imatinib monotherapy. Combination therapy also led to a decrease in Akt activation. Potent *in vivo* activity in animal experiments by combination therapy was noted, without toxicity to the animals. Our findings provide a rationale for the combined use of rapamycin and imatinib, both FDA approved drugs, for the treatment of TS.

## Introduction

TS is a common autosomal-dominant disorder characterized by the development of tumors of the brain, kidney, skin and lung. The disorder is characterized by mutations or deletions in one of two large genes, hamartin (*TSC1*) and tuberin (*TSC2*). In addition, neuro-developmental complications of TS, including seizures and autism, lead to significant morbidity. While no strict genotype-phenotype relationship has been established, the disease is more severe in patients with tsc2 mutations. Loss of heterozygosity (LOH), which deletes the unaffected allele, is required for the development of some neoplastic features of TS, including renal angiomyolipomas, lymphangiomyomatosis, and skin lesions, while LOH is not observed in brain tubers which often cause epileptic foci [1]. Apart from deletions, there are other mechanisms causing inactivation of tsc1 and tsc2.

The signaling pathways implicated in TS are complex. A hotspot for mutations in tsc2 involves regions implicated in controlling rheb, although multiple other signaling pathways have also been linked to TS-related neoplasia, including mTORC1, notch, p42/44 MAP kinase, NFkB, and Akt [2-11]. Tuberin (*TSC2*) was weak or absent in angiomyolipomas, but present in healthy kidney, whereas, phosphorylated p70 S6 kinase and pS6 were present only in angiomyolipomas. Activation of a mammalian target of rapamycin metabolic pathway in tuberous sclerosis lesions, which contributes to their growth [2]. The upregulation of mTORC1 observed in neoplasms of patients with TS has served as a rationale for recent use of mTOR inhibitor rapamycin (sirolimus) to treat TS. Rapamycin treatment was shown to result in partial regression of kidney, lung and skin lesions but not complete disappearance of tumors. In addition, tumor re-growth was observed upon cessation of therapy, consistent with the known cytostatic rather than cytotoxic effects of rapamycin. The use of mTOR inhibitors is becoming increasingly accepted, especially for the treatment in TSC [2,12,13]. These clinical findings suggest that additional signaling pathways are active in TS-related tumors [14].

We have previously demonstrated that platelet-derived growth factor β receptor (PDGFRβ) is present and active in human and murine TS lesions [15-17]. Other groups have demonstrated an inverse relationship between mTOR activation and PDGFRβ levels in TS-derived cells [18]. Therefore, we reasoned that mTOR blockade might be compensated for by PDGF activation in vitro and *in vivo*, and combined blockade might be more efficacious than rapamycin monotherapy. To test this idea, we used the FDA-approved drug imatinib (gleevec) to treat mouse tumors formed by implanted tsc2ang1 cells, isolated from a cutaneous sarcoma that arose in a tsc2 heterozygous mouse and a well-validated model of TS-related neoplasia. Imatinib functions as a specific inhibitor of a number of tyrosine kinase enzymes leading to downregulation of MMP-2 [19]. Currently, imatinib is approved for chronic myelogenous leukemia carring the bcr-abl translocation, as well as hypereosinophilic syndrome and eosinophilic leukemia with the FIP1L1-PDGFRα fusion kinase. Solid tumors for which imatinib is approved include c-kit positive gastrointestinal stromal tumor and inoperable dermatofibrosarcoma protuberans (www.gleevec.com). We demonstrate that addition of imatinib to rapamycin decreases the levels and phosphorylation of PDGFRβ. Consistent with this, combination treatment resulted in a greatly decreased phosphorylation of Akt, and Akt is a major determinant of tumorigenesis *in vivo*. Finally, we show that the combination of rapamycin and imatinib has a greater antitumor effect compared to vehicle alone than either rapamycin or imatinib compared to vehicle *in vivo*. These findings provide a rationale for combination therapy with rapamycin and imatinib in TS.

## Results

### Rapamycin and imatinib treatment inhibits TSC2 ang1 cell proliferation

**Figure 1:**
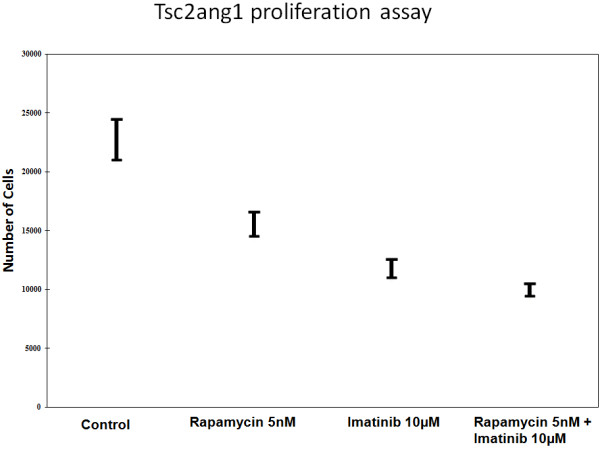
**(A) Effect of rapamycin, imatinib or combination of imatinib and rapamycin on tsc2ang1 cell line proliferation.** Cells were treated with vehicle control (DMSO), rapamycin (5nM), imatinib (10 μM) or imatinib (10 μM) + rapamycin (5nM) (shown along X-axis) for 24 h and counted using a Coulter counter. Treatment at each concentration was performed in triplicate. The Y-axis represents cell number. (**B**) Downregulation of Phospho-PDGFR-β by combinational treatment; Tsc2ang1 cells were treated with DMSO, rapamycin5nM, imatinib10uM or rapamycin5nM + imatinib10uM for 24 hours prior to harvest. Combination rapamycin + imatinib treatment inhibits the levels of phospho- PDGFR-β (Tyr 1021).

To assess if rapamycin and imatinib combination was superior to either agent alone, we assessd their effects on the proliferation of Tsc2 ang1 cells. Treatment of cells for 72 h with rapamycin (5nM) or imatinib (10 μM) alone reduced the level of proliferation significantly compared with control (p = 0.0006 for imatinib vs control, p = 0.0076 for rapamycin vs control). However, combined treatment with with both agents led to significantly better inhibition of proliferation (Figure 1a), compared with control (p = 0.0002). Combination of drugs was significantly better than rapamycin alone (p = 0.0243), while imatinib vs combination did not reach significance at p less than 0.05. Experiments were done in triplicates for reproducibility.

### Combination of rapamycin and imatinib inhibits PDGFRβ activation inTsc2 ang1 cells

In view of the effects of rapamycin and imatinib combination on Tsc2Ang1 cell proliferation together with previous findings that mTOR inhibitor treatment of TS is associated with increased PDGFRβ levels, we examined the effects of imatinib and rapamycin or a combination of these on PDGFRβ expression. Experiments were done in triplicates for reproducibility. We tested imatinib (10 μM), rapamycin (5nM) or their combination on the levels of phosphorylated PDGFRβ (at Tyr-1009) in Tsc2ang1cells. Combined treatment led to downregulation of PDGFRβ phosphorylation, (Figure 1b).

### Treatment with combination of rapamycin and imatinib down regulates phosphoAkt473 protein levels in Tsc2ang1 cells

**Figure 2:**
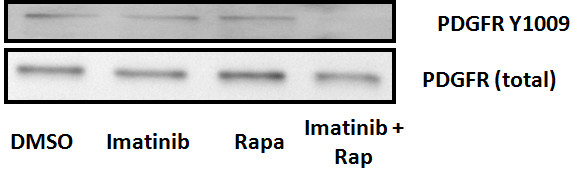
**blot analysis using Akt and pAkt were conducted on Tsc2 ang1 cells treated with treated with DMSO, rapamycin5nM, imatinib10uM or rapamycin5nM + imatinib10uM for 24 hours.** Cells were lysed and analyzed by using antibodies specific for the total Akt and pAkt473. Rapamycin + imatinib treatment downregulates pAkt levels (p <0.0002). GAPDH was used as the loading control by using monoclonal anti-GAPDH antibody. All treatment groups were significant when compared to the vehicle control, Imatinib 10nM (p < 0.0006); Rapamycin 5nM (p < 0.0076); Rapamycin 5nM + Imatinib 10nM (p < 0.0002). However, the combinational therapy only show significance against Rapamycin at 5nM (p < 0.0243) and not Imatinib at 10nM (p < 0.4273).

To verify the effect of imatinib and rapamycin, as well as combination therapy on the activation of Akt, we treated Tsc2ang1 cells with imatinib (10 μM), rapamycin (5 nM) or their combination and the levels of Akt and p Akt were measured by densitometry scanning of the signals on western blot membrane. Total levels of Akt were unchanged by treatment and served as loading controls. We found first that overall, treatment with drug led to significantly decreased phospho Akt compared to vehicle controls (p = 0.0002, general linear modeling). In addition, we found that treatment with rapamycin and imatinib reduced the levels of pAkt (Figure 2) and a significant reduction in the levels of pAkt (p <0.0002) in imatinib + rapamycin treated cells compared to vehicle control. The mean of combination treatment versus imatinib alone or rapamycin alone also decreased. However, significance was only observed when the combination treatment was compared to rapamycin alone (p < 0.0243) and not when compared to imatinib alone (p < 0.4273).

### Matrix metalloproteinase analysis

**Figure 3:**
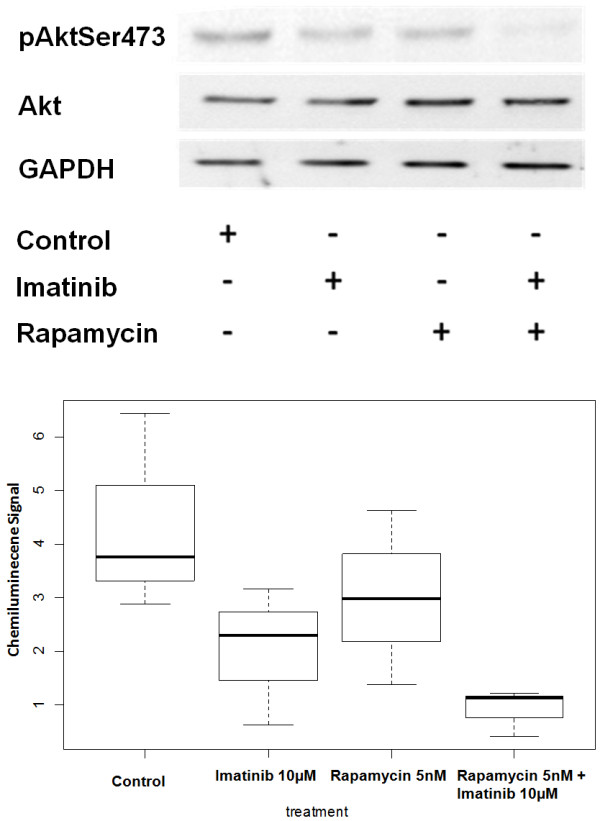
**MMP-2 ELISA analysis of conditioned media from tsc2ang1 cells treated with either rapamycin, imatinib or the combination for 24 hours resulted in a decrease in the amount of MMP-2 produced in comparison to controls.** MMP-2 levels in the tsc2ang1 cells treated with imatinib only and imatinib + rapamycin were significantly decreased compared to that of controls.

Cell migration and invasion are fundamental components of tumor cell invasion and neovascularization. Matrix metalloproteinases (MMPs) are essential for successful cell migration through extracellular matrix. In order to determine the levels of MMPs in our samples, conditioned media from equivalent numbers of imatinib- or rapamycin-treated cells along with that of control cells were collected and quantitatively analyzed using monospecific ELISAs. MMP-2 levels in conditioned media from tsc2ang1 cells treated with either imatinib (p = .0243), rapamycin or combination treatment with rapamycin and imatinib (p = .0092) were significantly decreased compared to that of controls (Figure 3). However, MMP-2 levels in the conditioned media of cells treated with rapamycin (p = .0744) were not significantly decreased compared to that of controls consistent with previous reports [20]. Differences between rapamycin alone and combination therapy trended toward significance (p = 0.0744), and between imatinib alone and combination (p = 0.329), indicating that imatinib has a more potent effect on MMP-2 activity than rapamycin. Moreover, no significant difference was observed in MMP-9 levels between the different treatment groups and controls (data not shown).

### *In vivo* tumorigenesis

**Figure 4:**
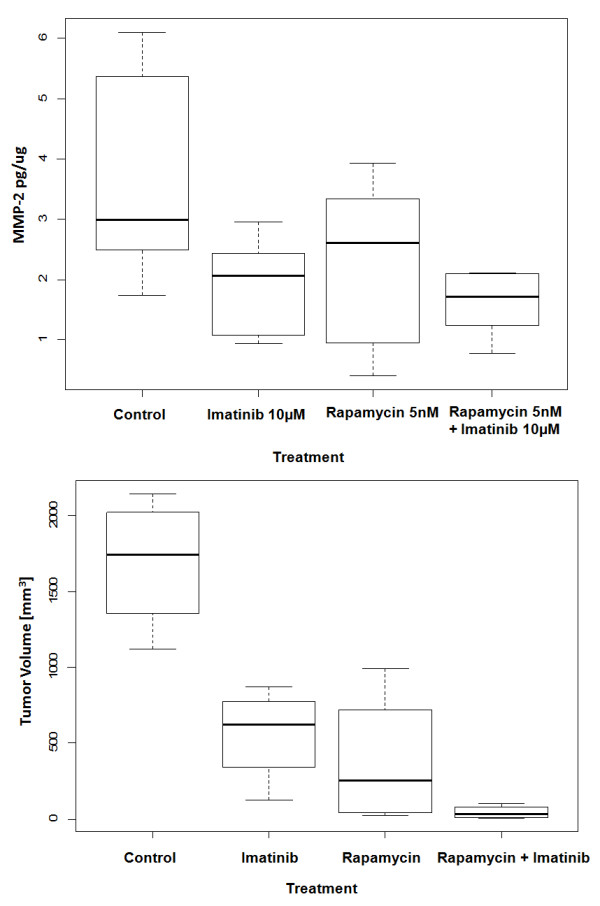
**Effect of rapamycin and imatinib on *in vivo* growth of tsc2ang1 xenografts in nude mice.** The y axis represents tumor volume.

Given the effects of combined treatment with imatinib and rapamycin on cell proliferation as well as the ability of this combination to reduce the levels of phospho-PDGFRβ and phosphor-Akt, we assessed the effects of this combination on tsc2ang1 tumorigenesis *in vivo.* While each drug individually reduced the size of tumors formed compared to vehicle control, combined treatment with imatinib and rapamycin resulted in a nearly complete abrogation of tumor growth (97% decrease in tumor volume compared with control, p < 0.0001) (Figure 4). Tumor volume was significantly different in comparison between control vs rapamycin/imatinib, rapamycin vs rapamycin/imatinib, and imatinib vs rapamycin/imatinib (p < 0.05). There was no significant difference between rapamycin alone and imatinib alone. Tumor volume comparing imatinib alone versus combination was significantly different (p = 0.0209), while tumor volume between rapamycin alone and combination did not reach significance (p = 0.19). Neither local nor systemic toxicity was observed in any of the treatment groups indicating that combined effective inhibition of tumor growth is feasible with imatinib and rapamycin with minimal toxicity.

## Discussion

TS is a multisystem disorder characterized by benign or malignant neoplasia, as well as autism and seizures. No highly effective and long lasting therapy exists for TS neoplasia. Renal lesions such as angiomyolipomas may cause massive bleeding and compromise renal function, often requiring a kidney transplant. Lymphangiomyomatosis, a neoplastic complication most commonly seen in young women, can only be cured by lung transplantation. Deforming skin lesions cause significant psychological distress [21]. Finally, brain tumors such as subependymal giant cell astrocytomas, can cause morbidity and mortality, and multiple tubers results in refractory seizures and exacerbation of mental retardation. Thus, urgent therapy is required for TS. Here, we demonstrate in a validated preclinical model a nearly complete tumor inhibition with a combination of two FDA-approved drugs (rapamycin and imatinib) targeting two distinct signaling pathways (mTORC1 and PDGFRβ, respectively) implicated in TS-associated neoplasia.

The lack of total blockade by rapamycin is consistent with the incomplete clinical response of tumors to systemic rapamycin, implying that either additional pathways are already present in these tumors, or are induced by rapamycin monotherapy. High among the candidates are the PDGFRβ mediated signal pathways, especially since mTORC1 activation and PDGFRβ signaling have been shown to have an inverse relationship [18].

Treatment with combination of rapamycin and imatinib led to downregulation of PDGFRβ in tsc2ang1 cells, in a c-cbl-independent manner (data not shown). In vitro proliferation assays demonstrated an additive effect of rapamycin and imatinib on tsc2ang1 cells.

Rapamycin has been used as monotherapy in patients with TS, resulting in benefit only as long as the patients are exposed to the drug [12]. Our findings that rapamycin therapy alone does not address PDGFβ signaling, as well as modest efficacy against Akt activation are potential reasons for the failure of rapamycin as monotherapy in solid tumors. In highly malignant tumors, mTORC1 inhibition has shown to cause a paradoxical activation of Akt, in part through activation of mTORC2, which is an Akt kinase [22,23]. We demonstrate that the combination of rapamycin and imatinib blocks Akt activation compared with monotherapy, and likely accounts for the decrease in tumor volume. Our findings demonstrate the following. Combination therapy of TS relevant cells with rapamycin and imatinib results in downregulation of PDGFRβ and Akt signaling. This decrease is independent of c-cbl-mediated degradation. Finally, the combination of rapamycin and imatinib has at least additive activity against the tsc2ang1 model of TS *in vivo*. Given that the combination of these two approved drugs is well tolerated *in vivo*, and that rapamycin alone does not cause long-lasting remissions, this study provides a rationale for the combination of these two drugs in humans.

## Materials and methods

### Generation of murine model of TS

Tsc2ang1 (ATCC CRL 2620) is a murine cell line derived from a cutaneous sarcoma that arose in the extremity of a mouse heterozygous for tsc2; these mice develop cutaneous sarcomas at a frequency of 10 to 15%. The cells were cultured in complete DMEM medium supplemented with 10% FBS (Sigma Aldrich, St Louis, MO).

### In vitro proliferation assay

10,000 tsc2ang1 cells per well were plated in 24-well dishes in triplicate. The next day, fresh medium containing the compounds or vehicle controls was added. Cells were incubated at 37° C for 24 h, and cell number was determined using a Coulter Counter (Hialeah, FL).

### Western blot analysis

Lysates of Tsc2ang1 cells treated with vehicle or indicated drugs were prepared in NP-40 lysis buffer (1% NP-40, 150 mmol/L NaCl, 10% glycerol, 20 mmol/L HEPES, 1 mmol/L phenylmethylsulfonyl fluoride, 2.5 mmol/L EDTA, 100 μmol/L Na_3_VO_4_, and 1% aprotinin). Protein concentration in cleared lysates was determined using an Eppendorf BioPhotometer. Samples were resolved using SDS-PAGE (National Diagnostics) and transferred to nitrocellulose membranes. The membranes were blocked with 5% nonfat dry milk in 10 mmol/L Tris/0.1% Tween 20/100 mmol/L NaCl and incubated with primary antibodies followed by horseradish peroxidase–conjugated secondary antibody. The immunoreactive bands were visualized by enhanced chemiluminescence (Amersham Biosciences). The antibodies used were: Phospho-PDGFR- β antibody(Tyr 1021) (Cell signaling Laboratories); Akt antibody (9272) (Cell signaling Laboratories), pAKT (4058) antibody (Cell signaling Laboratories) monoclonal anti-GAPDH antibody (Santa Cruz L-18 S-48167) was used as a loading control.

### Matrix metalloproteinase analysis

The presence of MMPs in conditioned media samples was determined using MMP ELISA Quantikine Kits (R&D Systems, Inc.). Specimens, standards and reagents were prepared according to manufacturer's instructions. Protein concentration was determined via the Bradford method using bovine serum albumin as the standard as described previously [24].

### *In vivo* tumor growth

To test the *in vivo* activity of compounds that inhibit tsc2ang1 growth in vitro*,* groups of four nude mice per compound (or control). We injected 1 million Tsc2ang1 cells s.c. into 4 nude mice in each group. I.p. treatment with imatinib, rapamycin and imatinib and rapamycin were conducted for 30 days. Rapamycin and imatinib were obtained from LC Laboratories (Woburn, MA). Beginning 2 days later, the mice received daily i.p. injections of vehicle (control), rapamycin (12 mg/kg/day), imatinib (120 mg/kg/day) or imatinib plus rapamycin. The compounds were suspended in 0.1 ml of ethanol and 0.9 ml of Intralipid solution (Fresenius Kabi, Uppsala, Sweden) [25]. No local or systemic toxicity was observed in any of the animals. Injections were given over a period of 4 weeks, after which the mice were sacrificed due to overwhelming tumor burden in the control group. Tumor volume was calculated using the equation (w^2^ xL)0.52, where w(width) represents the shortest diameter of the tumor.

### Statistics

One way ANOVA, and non parametric test were preformed for the tumor volume statistics. We did parametric analysis when the conditions for ANOVA were satisfied and in case where conditions are not satisfied and if the variables are not normally distributed, we conducted corresponding non parametric test and opted to present results for Wilcoxon test.

## Competing interest

United States Patent Application 20070078142 Inventor: Jack L. Arbiser, patent filed by Novartis Treatment of tuberous sclerosis associated neoplasms.

## Authors’ contribution

BG,LW, ASC,MYB, and MGA performed experimental studies. SC and EV performed statistical analysis. HB, MAM and JLA designed experiments and wrote the manuscript.

## Ethical approval

Animals studies were performed in compliance with the Emory IACUC.
